# BioSAXS Measurements Reveal That Two Antimicrobial Peptides Induce Similar Molecular Changes in Gram-Negative and Gram-Positive Bacteria

**DOI:** 10.3389/fphar.2019.01127

**Published:** 2019-09-26

**Authors:** Andreas von Gundlach, Martin P. Ashby, Jurnorain Gani, Paula Matilde Lopez-Perez, Alan Roy Cookson, Sharon Ann Huws, Christoph Rumancev, Vasil M. Garamus, Ralf Mikut, Axel Rosenhahn, Kai Hilpert

**Affiliations:** ^1^Analytical Chemistry - Biointerfaces, Ruhr-University Bochum, Bochum, Germany; ^2^Institute of Infection and Immunology, St. George’s University of London (SGUL), London, United Kingdom; ^3^TiKa Diagnostics Ltd, London, United Kingdom; ^4^Institute of Biological, Environmental & Rural Sciences (IBERS), Aberystwyth University, Aberystwyth, United Kingdom; ^5^Institute of Global Food Security, School of Biological Sciences, Queens University Belfast, Medical Biology Centre, Belfast, United Kingdom; ^6^Helmholtz-Zentrum Geesthacht, Zentrum für Material- und Küstenforschung GmbH, Geesthacht, Germany; ^7^Institute for Automation and Applied Informatics (IAI), Karlsruhe Institute of Technology (KIT), Karlsruhe, Germany

**Keywords:** antimicrobial peptide, mode of action, SAXS, MRSA, electron microscopy, *E. coli*

## Abstract

Two highly active short broad-spectrum AMPs (14D and 69D) with unknown mode of action have been investigated in regards to their effect against the Gram-negative bacteria Escherichia *coli* and the Gram-positive bacteria methicillin-resistant *Staphylococcus aureus* (MRSA). Minimal inhibitory concentration (MIC) measurements using a cell density of 10^8^ cfu/ml resulted in values between 16 and 32 µg/ml. Time-kill experiments using 10^8^ cfu/ml revealed complete killing, except for 69D in combination with MRSA, where bacterial load was reduced a million times. Small-angle X-ray scattering of biological samples (BioSAXS) at 10^8^ cfu/ml was applied to investigate the ultrastructural changes in *E. coli* and MRSA in response to these two broad-spectrum AMPs. In addition, electron microscopy (EM) was performed to visualize the treated and non-treated bacteria. As expected, the scattering curves generated using BioSAXS show the ultrastructure of the Gram-positive and Gram-negative bacteria to be very different (BioSAXS is not susceptible to the outer shape). After treatment with either peptide, the scattering curves of *E. coli* and MRSA cells are much more alike. Whereas in EM, it is notoriously difficult to observe changes for spherical Gram-positives; the BioSAXS results are superior and reveal strongly similar effects for both peptides induced in Gram-positive as well as Gram-negative bacteria. Given the high-throughput possibility and robust statistics, BioSAXS can support and speed up mode of action research in AMPs and other antimicrobial compounds, making a contribution toward the development of urgently needed drugs against resistant bacteria.

## Introduction

The World Health Organization (WHO) has classified antimicrobial resistance as one of the biggest threats to global health and food security. The extent of the threat requires action not only from researchers, but from governments and society. For example, antibiotics are misused in ton scale in agriculture for growth promotion or prevention of disease, but actions are taken to reduce this—for example, the European Union has banned the use of antibiotics for growth promotion in 2006. Currently, about 700,000 to 1,000,000 people die worldwide each year because of antibiotic-resistant infections. In the O’Neil Report, it is estimated that, by 2050, the numbers increase to 10,000,000 more people than are currently killed by cancer (https://amr-review.org/Publications.html). In this report, it was estimated that the additional healthcare cost worldwide for antibiotic-resistant infections will reach US$100 trillion. The situation might even intensify since the number of newly developed antibiotics is steadily declining. FDA approval of new antimicrobials has dropped to three new molecular entities (NME) in this decade.

Antimicrobial peptides (AMPs) are potential novel antimicrobial drugs with some much-desired features, including a low chance of developing drug resistance and fast acting, broad-spectrum activity including multi-drug resistant bacteria. So far, only a few have been investigated in clinical studies (Czaplewski et al., 2016; Greber and Dawgul, 2017). There are more than 3,000 natural and artificial peptides described (http://aps.unmc.edu/AP/main.php); the vast majority is cationic. Although they have an enormous variety of sequences and structures, they share certain common features. Cationic antimicrobial peptides are structurally diverse, typically between 5 and 50 amino acids in length with at least one excess positive charge due to lysine and arginine residues and contain hydrophobic amino acids. In the last decade, it became increasingly clear that antimicrobial peptides (natural and artificial) have very different modes of action, and to make it even more complex, they may have not only one but multiple targets (Le et al., 2017) (Brogden, 2005; López-Pérez et al., 2017). Some AMPs are shown to solely act on the membrane; however, several studies have demonstrated that peptides can bind and interrupt the processes of intracellular components, for example, bind to ATP and inhibit ATP-dependent enzymes (Ahmad and Laughlin, 2010; Hilpert et al., 2010) and have the ability to bind DNA (Sim et al., 2017) and inhibit protein synthesis (Mardirossian et al., 2018). For drug development in the field of antimicrobials, it is important to focus on compounds with different modes of action as compared with conventional antibiotics; otherwise, cross-resistance may occur very fast. With about 3,000 natural peptides available, selecting the right lead candidate is therefore important. In addition, for preclinical drug development, determination of the mode of action is an important part in order to move toward clinical trials. We have developed a new high-throughput method that can support the selection of new natural or artificial peptides and give first impression of possible mode of action (Von Gundlach et al., 2016a). In this study, we present for the first time that this method can be applied for Gram-positive and Gram-negative bacteria. In addition, we present for the first time that two broad-spectrum peptides induce very similar changes in both Gram-positive and Gram-negative bacteria. We have used two broad-spectrum peptides, peptide 69 and peptide 14. Peptide 69 is a direct optimized linear variant of the natural-occurring bovine peptide bactenecin (Hilpert et al., 2005). Peptide 14 is an *in silico*–optimized peptide based on different natural and artificial peptides (Ramón-García et al., 2013).

The overall peptide drug market for many different diseases and diagnostics is steadily growing; about 60 peptide drugs were approved, with 150 in active clinical trials; and it expected to further grow from US$14.1 billion in 2011 to US$25.4 billion in 2018 (Fosgerau and Hoffmann, 2015; Lau and Dunn, 2018). Demands for peptide drugs have led to (A) improved scale-up technologies, (B) new large-scale GMP certified manufacturing facilities, and (C) innovative drug administration regimes. These recent developments in peptide drugs have coincided with an increasing cost of novel non-peptide antibiotics, meaning that AMPs might soon become a viable economic option for urgently needed new antimicrobial drugs.

Small-angle X-ray scattering of biological samples (BioSAXS)—for example, proteins, is a powerful method for the characterization of both ordered and disordered structures in biological samples that provides information about the sizes and shapes ranging from a few kDa to GDa (Kikhney and Svergun, 2015; Chen et al., 2018). In the last decades, X-ray technology has matured to allow the study of protein crystals and proteins in solution down to atomic resolution. The short wavelength of the X-rays (<1Å) is the key for the success as it enables the probing of small structures. Third-generation synchrotron facilities and the advent of diffraction limited fourth-generation storage rings in the near future will provide exceptional brilliance that enables rapid data acquisition (Schroer et al., 2018). In conjunction with the latest generation of single photon-counting detectors and autosampler-based sample delivery systems, hundreds of samples can be measured per hour (Hajizadeh et al., 2018; Pernot et al., 2018).

Four years ago, we hypothesized that BioSAXS could be useful to discriminate the mode of action of antimicrobial compounds. Five conventional antibiotics with different modes of action, polymyxin B, and an antimicrobial peptide consisting of L-amino acids were selected. Using BioSAXS, changes in structures on the length scale between 3 nm and 120 nm within bacteria as consequence of treatment with antimicrobial substances were monitored. For this study, a Gram-negative bacteria (*E. coli*) was used and for comparison transmission EM was performed (Von Gundlach et al., 2016a). In conclusion, subtle structural intracellular rearrangements in the bacteria can accurately be probed across large bacterial populations (hundreds of thousands of bacteria) within seconds (Von Gundlach et al., 2016a, Von Gundlach et al., 2016b). Thus, in case of *E. coli*, novel compounds with unknown modes of action can be grouped according to their effect on the bacterial morphology, and new responses can be identified.

The aims of this study are (1) demonstrating that BioSAXS can be used for Gram-positive bacteria and (2) a comparison of modes of action of two antimicrobial peptides against Gram-positive and Gram-negative bacteria. An application of BioSAXS for Gram-positive bacteria would widen the usefulness of the method, especially given the harder to interpret EM images for spherical Gram-positive bacteria. The two selected peptides show broad-spectrum activity, and we wanted to compare their mode of action when killing Gram-positive and Gram-negative bacteria. Do the peptides have one mode of action for both classes of bacteria or one mode of action for each class? Since this is the first study of its kind, variability between treatments was kept to a minimum. There could be different expression level or different kinds of proteases that might lead to change in peptide concentration, and various fragments might possess different activities. In order to achieve proteolytic stability in high bacteria numbers, stereoisomers were used. D-peptide forms (14D and 69D) also showed broad-spectrum activity, and the time-kill experiments using the D-peptides demonstrated a fast-acting mode of action even with a high number of bacteria present.

## Materials and Methods

### Bacterial Strains

Bacterial strains used for antimicrobial activity testing in this project were methicillin-resistant *Staphylococcus aureus* (*S. aureus*) HO 5096 0412 (a neonatal infection isolate, isolated in Ipswich, England in 2005); a methicillin-sensitive *S. aureus* (ATCC 29213); *Escherichia coli* (*E. coli*, UB1005, F-, LAM-, gyrA37, relA1, spoT1, metB1, LAMR); *E. coli* (ATCC 25922); *E. coli* (68610Y); a clinical isolate from St. George’s University Hospitals NHS Foundation Trust, London, UK, resistant to gentamicin, ciprofloxacin, and ceftazidime, obtained from Timothy Planche; *Enterococcus faecalis* (*E. faecalis* ATCC 29212); a clinical isolate of *Staphylococcus epidermidis* (*S. epidermidis*) obtained from Dr. Robert E.W. Hancock (Department of Microbiology and Immunology, University of British Columbia); and vancomycin-resistant *E. faecalis* (NCTC 12203).

### Peptides

Antimicrobial peptides were synthesized by automated solid-phase peptide synthesis (SPPS) on a MultiPep RSI Peptide Synthesizer (INTAVIS, Tuebingen, Germany) using the 9-fluorenyl-methoxycarbonyl-tert-butyl (Fmoc/tBu) strategy. Reactive side chains were protected by *t*Bu (Tyr and Asp), trityl (Trt, for Asn, Cys, Gln, and His), 2,2,4,6,7 pentamethyldihydrobenzofuran-5-sulfonyl (Pbf, for Arg), and *tert*-butoxycarbonyl (Boc, for Lys and Trp). For automated SPPS, four equivalents of Fmoc amino acids (Bachem, Bubendorf, Switzerland) were coupled on TentaGel^®^ HL RAM resin (25-μmol scale, loading 0.3–0.4 mmol/g; Rapp Polymere, Tuebingen, Germany) after *in situ* activation with four equivalents of N,N,N′,N′-tetramethyl-O-(1H-benzotriazol-1-yl)uronium hexafluorophosphate (HBTU; Carbosynth, Berkshire, United Kingdom) and eight equivalents of N-methylmorpholine (NMM, Sigma, Dorset, United Kingdom). After double-coupling procedure (2x30 min), the Fmoc group was cleaved using 20% (*v*/*v*) piperidine (Thermo Fisher Acros Organics, Geel, Belgium) in dimethylformamide (DMF, Jencons-VWR, Leicestershire, United Kingdom). Peptide amides were cleaved from the resin with 95% (*v*/*v*) aqueous trifluoroacetic acid solution (TFA, Fisher Scientific, Loughborough, United Kingdom) containing 5% (*v*/*v*) triisopropylsilane (TIPS, Thermo Fisher Acros Organics, Geel, Belgium)/water (1:1) scavenger mixture within 3 h. Cleaved peptides were precipitated from ice-cold methyl *tert*-butyl ether (MTBE; Thermo Fisher Acros Organics, Geel, Belgium). After washing and collection by centrifugation, crude peptides were dissolved in 20% (*v*/*v*) acetonitrile (ACN, Jencons-VWR, Leicestershire, United Kingdom)/80% (*v*/*v*) water containing 1% (*v*/*v*) TFA to a concentration of 15 mg/ml and analyzed by analytical reversed-phase (RP) HPLC on a Shim-pack VP-ODS (120 Å, 150x4.6 mm, Shimadzu, Milton Keynes, United Kingdom) using a Shimadzu LC2010AHT system. The binary solvent system contained 0.1% (*v*/*v*) TFA in H_2_O (solvent A) and 0.1% (*v*/*v*) TFA in acetonitrile (solvent B). The identity was verified by a liquid chromatography electrospray ionization mass spectrometry (LC-ESI-MS) Shimadzu LC2020 system equipped with a Jupiter 4μ Proteo C18 column (90 Å, 250x4.6 mm, Phenomenex, Cheshire, United Kingdom). The binary solvent system contained 0.01% (*v*/*v*) TFA in H_2_O (solvent A) and 0.01% (*v*/*v*) TFA in acetonitrile (solvent B).

Crude peptides were purified to homogeneity of >92% by preparative RP HPLC on a Shimadzu LC2020 system equipped with a Jupiter 10μ Proteo C18 column (90 Å, 250x21.2 mm, Phenomenex) using a linear gradient system containing 0.01% (*v*/*v*) TFA in H_2_O (solvent A) and 0.01% (*v*/*v*) TFA in acetonitrile (solvent B). Pure products were finally characterized by analytical reverse phase high performance liquid chromatography (RP-HPLC) and liquid chromatography–mass spectrometry (LC-MS).

### Bacteriological Media and Culture Conditions

Mueller Hinton broth (MHb) (Merck) was used for all bacterial cultures. Media were prepared and sterilized according to the manufacturer’s’ instructions. Cultures were incubated at 37°C for 18–20 h with aeration, and cultures on solid media were incubated at 37°C for 18–24 h.

### Minimal Inhibitory Concentration Determination

Minimum inhibitory concentrations (MIC) were determined using a broth microdilution assay as previously described (Wiegand et al., 2008). Bacteria from an overnight culture grown at 37°C were diluted in fresh MHb to achieve a concentration of 1 x 10^6^ CFU/ml. A bacterial suspension (100 µl) was added to wells in a 96-well polypropylene microtiter plate that had been preloaded with serial dilutions of antimicrobial peptides in MHb (100 µl) giving a final bacterial concentration of 5 x 10^5^ CFU/ml. Microtiter plates were incubated at 37°C for 18–20 h before the MIC was determined as the lowest concentration of antimicrobial able to inhibit visible growth.

To determine the MIC toward 10^8^ CFU/ml (MIC_10^8_), which was the bacterial concentration used in BioSAXS experiments, bacteria from an overnight culture were diluted 1:100 in fresh MHb and incubated in a shaking incubator at 37°C and 250 RPM until an OD_600_ of 0.25 was reached, which equated to approximately 2 x 10^8^ logarithmically growing CFU/ml. The MIC was then performed as above without a further dilution of the culture. After 18–20-h incubations, 10 µl of a 500 µM resazurin solution (Sigma–Aldrich) were added to each well of the microtiter plate, and the cell viability was determined after a further 1-h incubation by the colorimetric reaction that occurs in the presence of viable cells.

### Time-Kill Curves

Overnight cultures of MRSA and *E. coli* UB1005 were diluted 1:100 in MHb and placed in a shaking incubator at 37°C until an OD_600_ of 0.25 was reached. The culture was then diluted in MHb to achieve a concentration of ∼1 x 10^8^ CFU/ml; this culture was split into 1.5-ml tubes. Antimicrobial peptides were added at concentrations of 2 x MIC, and sterile water was used as a negative control. Samples were then placed in a shaking incubator set to 37°C. After 0, 10, 20, 40, 60, and 240 min, 20 µl of the sample was removed and 10-fold serial dilutions in 10-mM Tris buffer were performed to 10^−6^. From each of the dilutions, 5 x 5 µl was plated onto Mueller Hinton agar. Agar plates were placed in a 37°C incubator, and colony-forming units (CFUs) were counted after 24-h incubation.

### Sample Preparation for Biosaxs

A 300-µl aliquot of an overnight culture of MRSA or *E. coli* UB1005 culture was diluted 1:100 in MHb and placed in a shaking incubator set to 37°C until an OD_600_ of 0.25 was reached. The cultures were then aliquoted into several 2-ml clear plastic vials, and doses of peptide were added to achieve a final concentration 2 x MIC_10^8_ (MIC determined for 10^8^ CFU/ml). Additional vials containing culture, but no drug, were included as negative controls. The vials were placed in a shaking incubator (250 rpm) at 37°C and incubated for 40 min. Each sample was then washed twice by centrifugation (SciQuip Ltd., UK) at 10,000 RPM for 5 min. Each wash involved the supernatant being removed and pellet resuspended in 1 ml 0.1 M PIPES buffer (pH 7). Samples were then centrifuged for a third time, and the pellet was resuspended in 1 ml of 2.5% glutaraldehyde v/v in PIPES buffer. The samples were then shaken at room temperature for 1 h and then washed three times in PBS buffer; at the end of the final washing, step the pellet was resuspended in 100 µl of PBS. All samples were then refrigerated at 5°C before analysis.

### Small-Angle X-Ray Scattering

The small-angle scattering experiments were performed at the BioSAXS beamline P12 at PETRA III (EMBL/DESY) in Hamburg, Germany as in previous experiments. A photon flux of 5 x 10^12^ s^−1^ focused to a spot size of 0.2 mm x 0.1 mm (horizontal x vertical) and the resulting diffraction pattern were recorded with a Pilatus 2M detector (Dectris, Switzerland). The sample (20 µl) was delivered into a cooled glass capillary (20°C) by an automated sample robot. For each sample, 20 diffraction patterns were recorded with an exposure time of 0.05 s. Before and after every sample, the background was measured. After angular integration to obtain one-dimensional scattering curves, the background subtraction was performed. To avoid introduction of artifacts by radiation damage, curves collected in subsequent illuminations are compared by a standard F-test (Franke et al., 2012). Only curves collected before the occurrence of radiation damage were further processed. This primary data processing steps were performed using the automated data pipeline SASFLOW.

### Data Evaluation

Scattering data was analyzed using the open-source data mining MATLAB^®^ Toolbox Gait-CAD and its successor SciXMiner, using the “Peptide Extension” tool (Mikut, 2010; Mikut et al., 2017). At first, the first data points afflicted by beamstop were removed. To compensate for the experimental variation of the cell density, the data was normalized to the initial region (0.04 to 0.05 nm^−1^). In order to be consistent with our former data, we decided to perform a PCA even with fewer data from these experiments. For the principal component analysis (PCA), the log of the scattering data was used and the range had to be restricted (0.055 to 0.2869 nm^−1^) due to low intensity of several scattering curves. The PCA is an easy visualization that preserves the main differences of the investigated scattering curves. The sample points are projected to a lower dimensional parameter space, built by so-called principal components. These principal components are orthogonal to each other and remove the redundancies caused by correlations of the sample points. They are computed by finding the eigenvalues of the covariance matrix of the 94 data points per scattering curve. The SAXS data were measured in the q-range of 0.02 and 4.8 nm^−1^. The 94 data points are contained in the q-range between 0.055 and 0.2869 nm^−1^ which was used in PCA analysis (**Figure 3**). The first two principal components were found to describe the variations due to antibiotic treatments. For reasons of better visualization, a centered PCA starting from the mean of all scattering curves *I* *_m_*(*q*) was used: *I*(*q*) = *I*_m_(*q*)+*A*·*PC1*(*q*)+*B*·*PC2*(*q*). Consequently, each scattering curve can be approximated by two linear coefficients (*A*, *B*). To provide evidence of reproducibility between two measurements, we measured duplicates of a subset of samples. The mean of the two measurements was used for further analysis. The experimental error estimate given was calculated as average standard deviation of all repeats.

### Electron Microscopy

MRSA and *E. coli*, untreated and treated with 14D and 69D for 40 min, were subjected to an ethanol series of 30, 50, 70, and 95% and three changes of 100% for at least an hour. The samples were transferred to a 1:2 mixture of ethanol to LR White—Hard Grade (London Resin Company, UK) resin then a 2:1 mixture of ethanol to resin and finally 100% resin overnight at 4°C. The next morning, the resin was removed and replaced with fresh resin, and later that day, the samples were placed in size four gelatine moulds (Agar Scientific), filled with fresh resin, and polymerized overnight in an oven at 60°C. Two-micrometer thick sections were cut which contained the bacteria, and these were dried in drops of 10% ethanol on glass microscope slides. They were stained with AMB stain (azur II and methylene blue, both Sigma-Aldrich Ltd., UK) and photographed using a Leica DM6000B microscope. Ultrathin 60–80-nm sections were then cut on a Reichert-Jung Ultracut E Ultramicrotome with a Diatome Ultra 45 diamond knife and collected on Gilder GS2X0.5 3.05-mm diameter nickel slot grids (Gilder Grids, Grantham, UK) float-coated with Butvar B98 polymer (Agar Scientific) films. All sections were double-stained with uranyl acetate (Agar Scientific) and Reynold’s lead citrate (TAAB Laboratories Equipment Ltd., Aldermaston, UK) and observed using a JEOL JEM1010 Transmission Electron Microscope (JEOL Ltd., Tokyo, Japan) at 80 kV. The resulting images were photographed using Carestream 4489 Electron Microscope Film (Agar Scientific, UK) developed in Kodak D-19 developer for 4 min at 20°C fixed, washed, and dried according to the manufacturer’s instructions. The negatives were scanned with an Epson Perfection V800 film scanner and converted to positive images.

## Results

**Figure 1:**
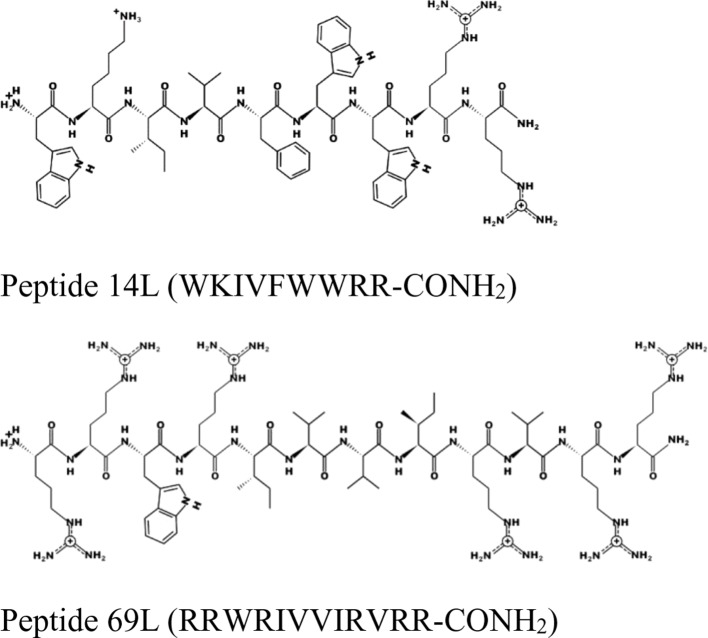
Schematic representation of the peptides 14L and 69L using the program PEPDRAW (http://pepdraw.com/).

For this experiment, two broad-spectrum short antimicrobial peptides were selected, which were previously described (Hilpert et al., 2005; Ramón-García et al., 2013) (see **Figure 1**).

Peptide 69L (RRWRIVVIRVRR-CONH_2_) is an all-L amino acid peptide comprising of 12 amino acids. Peptide 69L is an optimized variant of bactenecin (RLCRIVVIRVCR-CONH2), which is produced in bovine neutrophils as a 155-mer precursor polypeptide, containing a pro-region responsible for translocation of the peptide into granules, in which the mature bactenecin concentration is estimated to reach ∼12mg/ml (Romeo et al., 1988; Storici et al., 1992). The peptide demonstrated modest antibacterial activities against the human Gram-negative *E. coli* and Gram-positive *S. aureus* pathogens (Gallis et al., 1989–1990; Romeo et al., 1988; Wu and Hancock, 1999). Linear variants of bactenecin were synthesized, and especially Bac2A (RLARIVVIRVAR-CONH2) showed promises for further development since it demonstrated antimicrobial activity and very low hemolytic activity (Wu and Hancock, 1999). The peptide Bac2A was further optimized by creating a substitutional analysis using the SPOT synthesis method (Frank, 2002; Hilpert et al., 2005; Hilpert et al., 2007), creating the peptide 69L. Peptide 14L (WKIVFWWRR-CONH_2_) is an all-L amino acid peptide comprising of nine amino acids and was predicted *in silico* based on different natural and artificial peptides (Ramón-García et al., 2013). Both peptides are amidated at their C-terminus. MIC values for these peptides against a series of human pathogens are given in **Table 1**. That includes methicillin-sensitive and methicillin-resistant *S. aureus* (Gram-positive), vancomycin-sensitive (ATCC 29212) and vancomycin-resistant (NCTC 12203) *E. faecalis* (Gram-positive), *Staphylococcus epidermitis* (Gram-positive), and three *E. coli* (Gram-negative) strains—a recommended reference strain for antibiotic susceptibility testing (ATCC 25922), a typical laboratory strain (UB 1005), and a highly resistant clinical isolate (68610Y). Both L-peptides show a broad-spectrum activity against these strains. There is no difference between the resistant and sensitive variants, showing that the enquired mechanism of resistance is not effective against these antimicrobial peptides, indicating a different mode of action as the clinically used antibiotics. Since the BioSAXS experiment requires 1,000 times higher bacterial concentrations than are used in classical MIC tests, we decided for this particular experiment to use the all-D peptides in order to avoid problems with fast proteolytic attack by bacterial proteases. These all-D peptides are called 14D and 69D. The transformation of an all L-peptide into an all D-peptide did not affect the MIC, indicating the same mode of action is still conserved. Since the activity was very similar against these various strains, and in order to keep the comparison simple, one Gram-positive (MRSA) and one Gram-negative bacteria (*E. coli* UB1005) were selected for further investigation.

**Table 1:** Minimal inhibitory concentration (MIC) in µg/ml for four peptides against several Gram-positive and Gram-negative bacteria. All MICs were performed in Mueller–Hinton bouillon at least three times, and data are stated as the modal value. MRSA stands for methicillin-resistant *Staphylococcus aureus* and VRE for vancomycin-resistant *Enterococcus faecalis*.

Peptide/*bacteria*	Staphylococcus aureus ATCC 29213	MRSA	Escherichia *coli* ATCC 25922	Escherichia *coli* UB1005	Escherichia *coli* 68610Y	VRE NCTC 12203	*Enterococcus* faecalis ATCC 29212	*Staphylococcus* epidermidis
**T14L**	0.5	0.5	2	4	2	1	1	<0.25
**T14D**	1	1	2	2	2	1	1	<0.25
**T69L**	2	2	2	2	2	2	4	0.5
**T69D**	2	1	2	2	2	2	2	0.5

**Figure 2:**
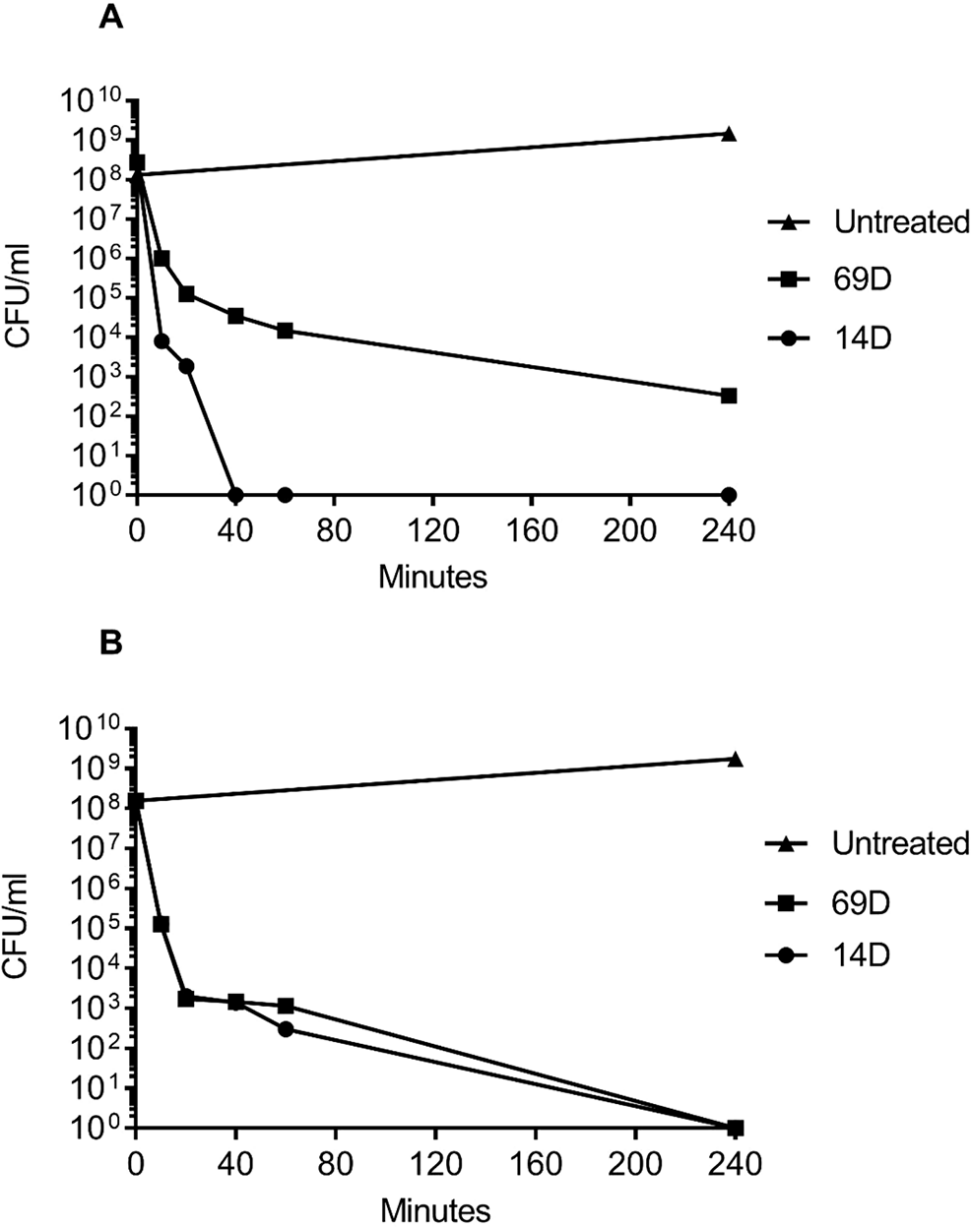
Time-kill curves of **(A)** MRSA and **(B)** *E. coli* following incubation with the antimicrobial peptides 14D and 69D in Mueller Hinton broth. Peptides were used at twice the MIC required to inhibit the growth of 1 x 10^8^ CFU/ml for each organism.

For a MIC determination, an inoculum of about 2−5x10^5^ bacterial cells is used; however, for the BioSAXS, a bacterial density of about 1x10^8^ cells is required, and in consequence, more peptide molecules are needed in order to kill or inhibit bacterial growth. The MIC for both peptides with an inoculum size of 10^8^ was determined with 26 µg/ml for 14D and 32 µg/ml for 69D against *E. coli*, and 16 µg/ml for 14D and 32 µg/ml 69D against MRSA. For the time-kill assay, twice the MIC_10^8_ concentration was used. Both the peptides were able to kill 1x10^8^ bacterial cells completely, except for 69D against MRSA, where the bacterial load was reduced a million times (see **Figure 2**). In case of MRSA, peptide 14D killed all bacteria after 40 min, indicating an “end point” to the mode of action. In consequence, 40 min was used as a time point for BioSAXS as well as EM. The BioSAXS method will detect all ultra-structural changes, stemming directly from the action of the antimicrobial compound as well as from the bacterial response to the compound. For both peptides, a significant killing occurs in the first 10 min, and structural changes induced by the bacteria might be still minimal. Therefore, 10 min of incubation time was used as an additional time point for the BioSAXS experiment. In the BioSAXS experiment, hundreds of thousands of bacteria can accurately be probed across large bacterial populations resulting in a robust statistic. Independent samples were used to perform double measurements to exclude artifacts. The sample size for each condition was n = 2.

**Figure 3:**
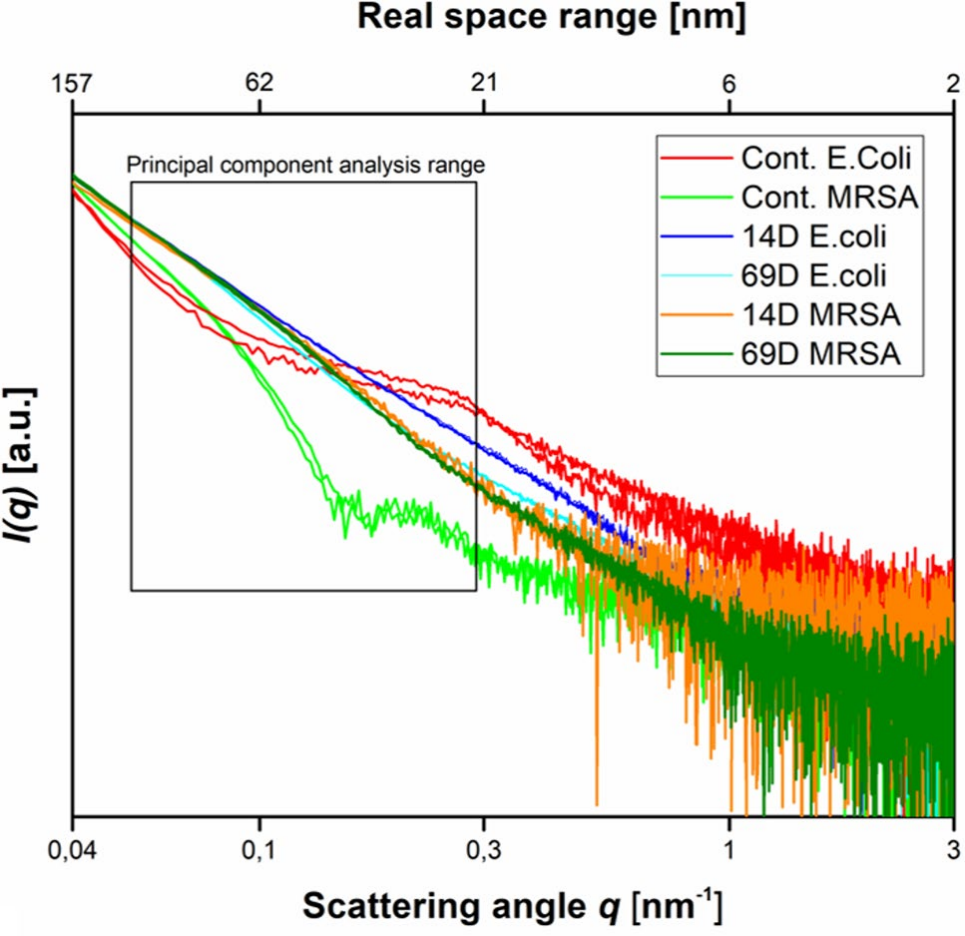
Scattering data as measured at the P12 BioSAXS beamline at PETRA III (Hamburg, Germany) at a photon energy of 10 keV. Scattering data from *Escherichia coli*, MRSA untreated (Cont. *E. coli* and Cont. MRSA) and treated with peptides 14D and 69D (in color code) at 40 min measured in duplicate (shown as separate curves). The box indicates the range that was used to calculate the PCA.

Using two times the MIC_10^8_ (required to inhibit the growth of 1 x 10^8^ CFU/ml) and 10^8^ cells, peptides 14D and 69D were incubated with the bacteria, and after 10 and 40 min, samples were taken to be processed for BioSAXS measurement and at 40 min for EM (see *Materials and Methods*). The results of the scattering are shown in **Figure 3**.

**Figure 4:**
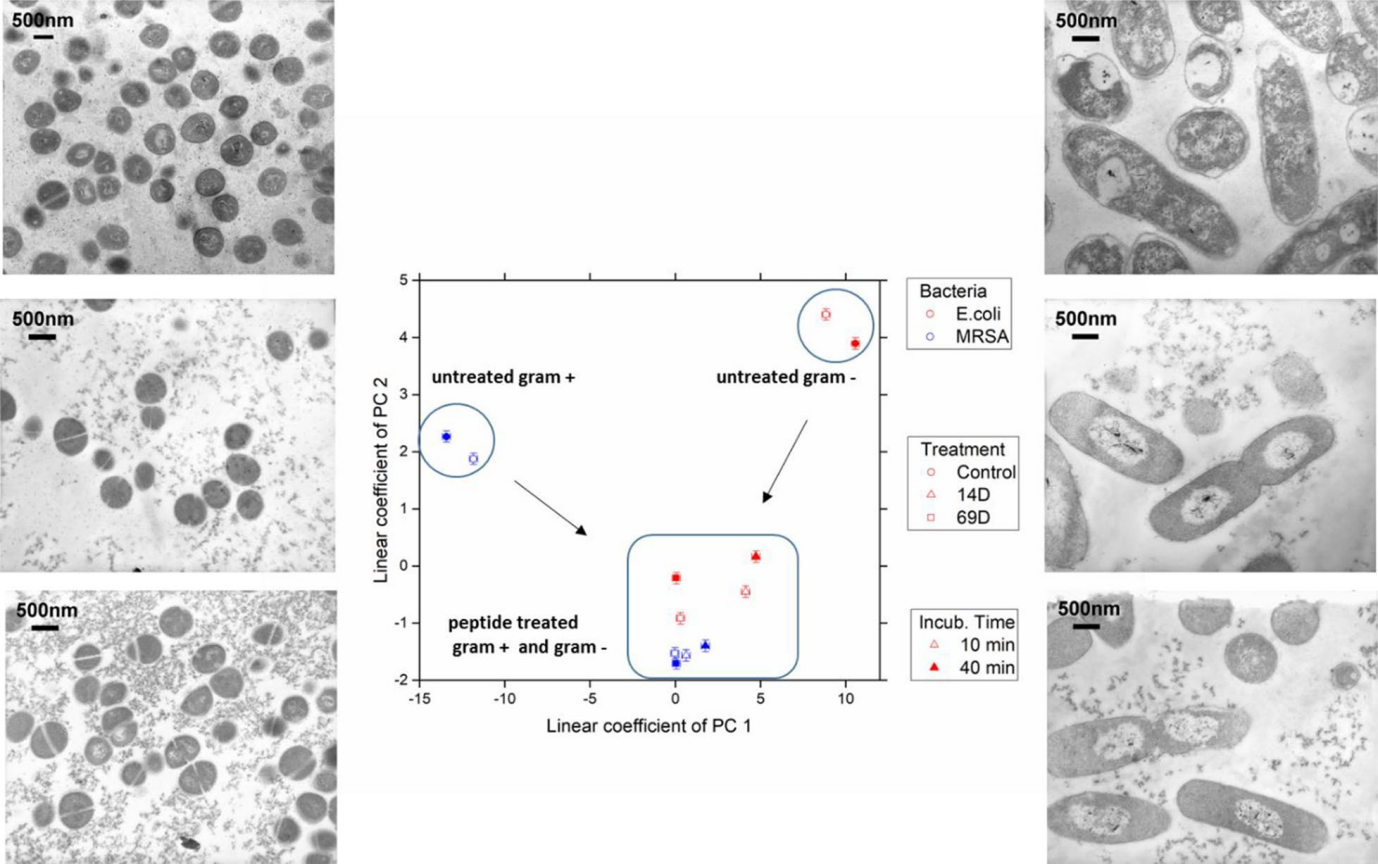
The linear coefficients of the first two principle components discriminate morphological changes and modes of action. The color decodes the bacterial species, the symbol the applied treatment, and the symbol thickness the incubation time. The error estimate is calculated from duplicate measurements and found to be 0.24 for the coefficient of PC1 and 0.10 for the coefficient of PC2. The transmission electron micrographs at a 15,000 times magnification show MRSA left and *Escherichia coli* right hand side. Top row shows untreated cells; middle row, treatment with 14D; and lower row, treatment with 69D. Peptide treatment time was 40min.

With respect to the size range covered by the small-angle X-ray scattering experiments, untreated cells of *E. coli* and MRSA differ mainly between a size range of 20 to 60 nm, with higher contribution from the Gram-negative cell. The measurement is only susceptible to the internal structure and not the outer shape of the bacteria. After treatment with either peptide, the scattering curves of *E. coli* and MRSA cells are much more alike—smoother with a constant slope. In order to better visualize the differences in the scattering curves, a principle component analysis was performed using the curve section shown in **Figure 3**. The result of this analysis and the EM images of *E. coli* and MRSA at 40 min are presented in **Figure 4**.

At the PCA plot, untreated MRSA (left side and middle of the plot) occupies a very different space than untreated *E. coli* (right side, upper part) with PC 1 as the main discriminator. Already after 10 min of treatment, data from both MRSA and *E. coli* are more similar. Especially MRSA data is very similar to each other and does only change slightly when comparing 10 and 40 min. For *E. coli*, where a slower killing kinetic was observed, changes between 10 and 40 min are more pronounced. For more information, see *Discussion*. The transmission electron microscopy (TEM) images of *E. coli* after 40 min show a clear difference between treated and untreated cells. In particular, the cytosol is less structured, and the nucleoid is collapsed into the center and enlarged (see *Discussion*). TEM images of MRSA also show differences in the structure of the cytosol.

## Discussion

Antimicrobial peptides are potential novel antimicrobial drugs with some much-desired features—for example, a low chance for the development of resistance, fast-acting, broad-spectrum of activity and activity against multidrug resistant bacteria. There is a large variety of structures and sequences of AMPs and, in recent years, it has become clear that there are also a variety of targets (Le et al., 2017). Today, there is a huge body of literature regarding AMPs; however, there is only few articles published on target validation and pharmacological and safety studies (Schmitt et al., 2010; Czaplewski et al., 2016; Greber and Dawgul, 2017; Mardirossian et al., 2018). This contributes to the fact that only a few AMPs are enrolled in clinical studies. We have already shown that BioSAXS can support research on antimicrobials to select compounds with possible new modes of action and therefore select compounds with alternative mode compared with mechanisms of action of failing conventional antibiotics (Von Gundlach et al., 2016a). In this study, we compared the effects on Gram-positive and Gram-negative bacteria to further understand the broad-spectrum activity of two antimicrobial peptides.

The effect of an antimicrobial compound on bacteria can be quite complex. The compound will act on their target(s) and induces changes at this site which can lead to secondary effects at the same, or at different sites. In case the target(s) are inside the bacteria, the compounds will cross the outer envelopment and the membrane and could therefore cause additional changes. At the same time, the bacteria react to the compound and induce several stress responses and coping mechanism in order to survive. The observed effect is consequently a mixture of ultrastructural changes on the bacterial level caused by direct and indirect effects of the antimicrobial compound as well as direct and indirect effects of the stress response of the bacteria. For each compound, these effects will be concentration and time-dependent (Von Gundlach et al., 2016a).

The BioSAXS measurements require a high bacterial density, and therefore, higher amounts of proteases are present as compared to a conventional MIC test. The proteases could cleave the L-peptides into many different fragments which may render inactive or also interact with the bacteria and prompt a detectable alteration in ultrastructure. To restrict this, the L-peptide sequences were synthesized as complete D-versions that will be extremely stable in the presence of the proteases for the time frame of the experiment. For the BioSAXS experiment, only the complete D-versions were used; therefore, MIC values and time-kill assays were performed using complete D-peptides.

The EM images show that the treatment of *E. coli* with either peptide results in a separation of the cytoplasm and the nucleoid, which appears to be in the center of the cell. In addition, the cytoplasm becomes much more homogenous as compared to the control. Interestingly, the peptides in this study result in a very different response compared to a peptide (RLKRWWKFL) described in our previous studies, indicating different modes of action (Von Gundlach et al., 2016a). In addition, we could not detect any similarities, for example, damages to the cell wall or membrane and dramatic changes in the inside of the cells, typically seen with polymyxin B, a cyclic lipopeptide with detergent-like mode of action (Von Gundlach et al., 2016a). The type of nucleoid separation observed after a treatment with peptide 14D and 69D are similar to the ribosome-acting drugs such as chloramphenicol or tetracycline which may indicate a similar target or cell response (Von Gundlach et al., 2016a). From studies on living cells, it is known that an inhibition of the peptide synthesis leads to a compaction of the bacterial nucleoid while an inhibition of the RNA synthesis by rifampicin expands the bacterial nucleoid (Chai et al., 2014). The mechanism for the condensation of the nucleoid is described as the absence of “transertion,” the synthesis of membrane proteins in close proximity to the cell wall. When the protein synthesis is inhibited, the DNA/RNA complexes are no longer tethered to the cell wall which leads to a collapse of the nucleoid in the cell centre (Cabrera et al., 2009). Due to the spherical structure and high cell wall density of *S. aureus*, changes in the EM images between treated and untreated cells are harder to detect, although treatment with the peptides does also seem to lead to a more homogenous cytoplasm.

The scattering curves generated using BioSAXS show the ultrastructure of the Gram-positive and Gram-negative bacteria to be very different as expected. However, following treatment with both antimicrobial peptides 14D and 69D, the ultrastructure of the MRSA and the *E. coli* became more similar to each other. Drastic change occurs in the range of 20 to 45 nm. The average protein is between 2 and 10nm (large proteins like IgG about 10 nm), large protein complexes like ribosomes are about 20–30nm, and compacted protein/DNA complexes are about 30nm. This data in conjunction with the EM also indicates that ribosomes can be affected by the treatment as well as changes in the nucleoid. The ultracellular effect of 14D and 69D on *E. coli* is similar direction although not the same. The SAXS data reveal a structural difference in the first principle component. While both feature a condensed nucleoid, 69D also seems to affect the cellular wall. In MRSA, both 14D and 69D initiate very similar changes even after 10 min, which remain unchanged after 40 min. In *E. coli*, the strong morphological effect of the peptide already comes into play after 10 min. After 40 min, the alteration does not increase, rather reaching an equilibrium state.

In conclusion, so far, we had only shown that BioSAXS can be used as a method to study effects of antimicrobials on Gram-negative bacteria; here, for the first time, we show that Gram-positive bacteria can also be used to detect changes after peptide treatment. Whereas in EM, it is notoriously difficult to observe changes for spherical Gram-positives; the BioSAXS results are superior and reveal strongly similar effects for both peptides induced in Gram-positive as well as Gram-negative bacteria. Given the high-throughput possibility and robust statistics, we believe that BioSAXS can support and speed up mode of action research in AMPs and other antimicrobial compounds, making a contribution toward the development of urgently needed drugs against MDR bacteria.

## Data Availability Statement

All datasets generated for this study are included in the manuscript/supplementary files.

## Author Contributions

AG, CR and VG performed the BioSAXS measurement. AG, CR and RM analysed the data. RM provided a program for data handling. MA, JG, and PL-P performed MIC studies, time-kill experiments and peptide synthesis, purification and characterization. AC performed the transmission electron microscopy, SH supported this work. KH, AR and SH contributed conception and design of the study. AG and KH wrote the manuscript.

## Funding

KH thanks the St George’s University of London for a start-up grant. RM was funded by the BIFTM program of the Helmholtz Association. The work was funded by the Virtual Institute VH-VI-403 (Helmholtz Society) and the BMBF project 05K16PC1. The Deutsche Forschungsgemeinschaft and the Open Access Publishing Fund of Karlsruhe Institute of Technology funded the open access publishing fee.

## Conflict of Interest

The authors declare that one peptide described in this manuscript is patented (WO2013053772). PL-P was employed by company Tika Diagnostics Ltd. KH is a Director of Tika Diagnostics Ltd.

The remaining authors declare that the research was conducted in the absence of any commercial or financial relationships that could be construed as a potential conflict of interest.
